# Supplementary material for: Rapid remodeling of NTP levels enables immediate translational adaptation to energy stress in yeast
Source: Mol Cell. Author manuscript; Available in PMC 2026 Feb 3. (PMC7618711; doi:10.1016/j.molcel.2025.08.031)
Supplement: Supplementary Material [file EMS212089-supplement-Supplementary_Material.zip › 1-s2.0-S1097276525007361-mmc1.pdf]

Molecular Cell, Volume *85*

Supplemental information

Rapid remodeling of NTP levels enables immediate translational adaptation to energy stress in yeast

Katherine Bexley, Michaela Ristová, Sushma Sharma, Christos Spanos, Andrei Chabes, and David Tollervey

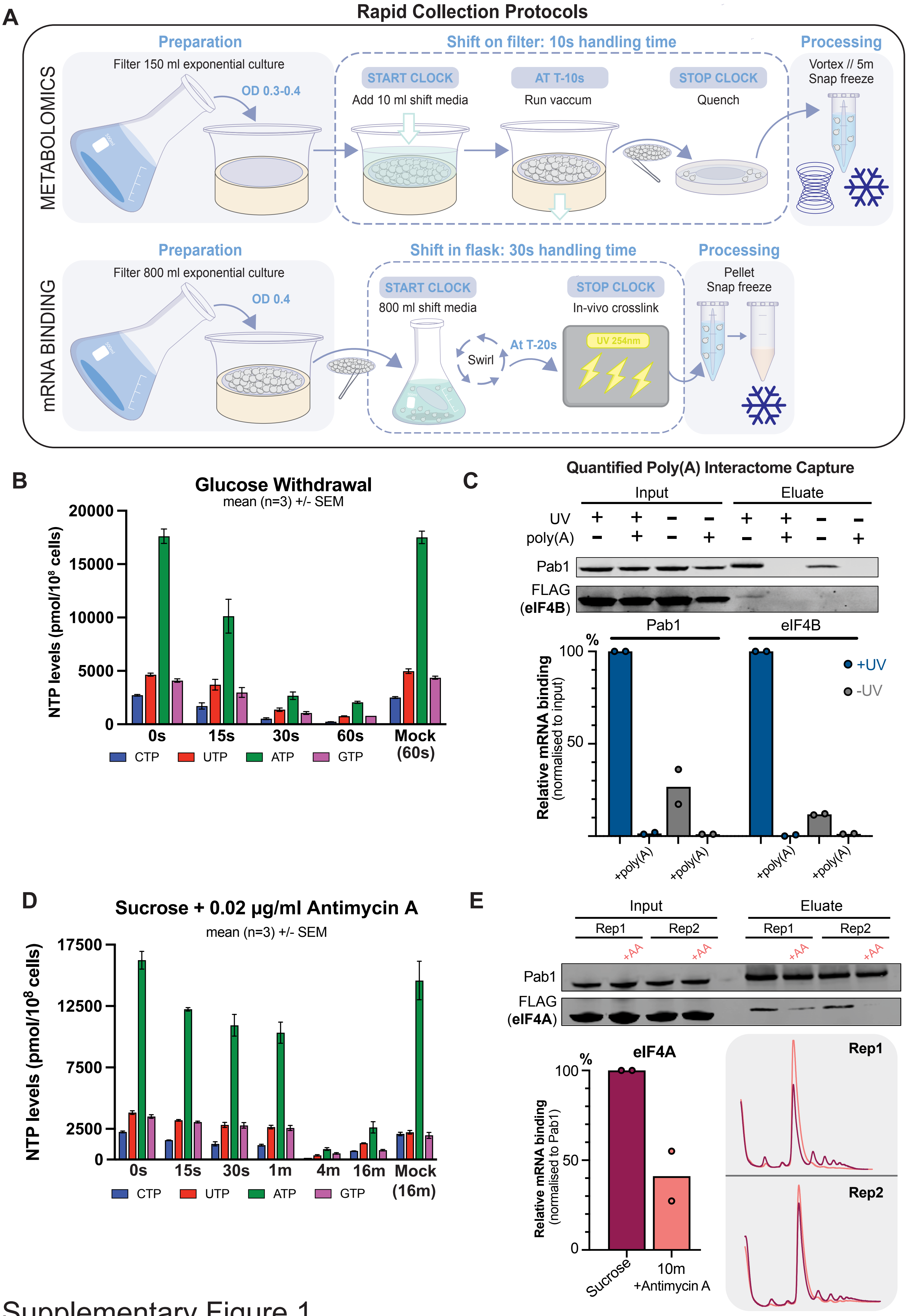

Supplementary Figure 1

Supplementary Figure 1. **Sample preparation and effects of glucose withdrawal** (related to Figure 1)

A: Schematic overview of the processes used for rapid carbon-source shift experiments. For metabolomics, exponential cultures were harvested by vacuum filtration. The fresh medium was applied while the yeast was retained on the filter and incubated for the indicated time. 10 sec was required to remove media and transfer the filter to lysis solution. In poly(A) interactome capture experiments, filtered cells were transferred to a fresh flask of shift media, resuspended and incubated shaking. The shifted culture was UV crosslinked at 254nm to stabilize RNA-protein interactions. 20 sec was required for handing and crosslinking. Yeast was then re-harvested for processing. Steps following crosslinking are less time-critical, as the covalent bonds have already been generated.

B: Bar chart showing NTP levels measured following glucose withdrawal in *S.cerevisiae.* Replicate samples (n=3) were obtained at 15 sec, 30 sec and 1 min following shift from 2% glucose to 2% glycerol/ethanol or a mock shift back to glucose. Error bars represent the mean +/− standard error (SEM).

C: Assessment of poly(A)-interactome capture specificity using non-crosslinked and poly(A) competed controls. Exponential glucose grown cultures were either UV crosslinked at 254 nm for 12s (+UV) or left untreated (-UV) prior to denaturing cell lysis. Diluted lysates (15mg/ml) were either input directly to pulldowns or prepared with artificial poly(A) (0.4mg/ml) as a competitor. The amount of Pab1and FLAG-tagged eIF4B purified and eluted in each case was assessed by western blotting (upper) and quantified by intensity relative to the input level (n = 2). Bar graphs (lower) represent the amount of Pab1 or eIF4B obtained in the eluate (mRNA-bound) as a percentage of the input level. UV treated samples are shown in blue, compared to untreated samples in grey.

D: Bar chart showing NTP levels measured following Antimycin A (AA) treatment in yeast grown on sucrose. Replicate samples (n=3) were obtained at 15 sec, 30 sec, 1 min, 4 min and 16 min following shift to 2% sucrose media containing 0.02 µg/ml AA or a mock return to 2% sucrose. Error bars represent the mean +/-standard error (SEM).

E: Simultaneous assessment of eIF4A-mRNA binding and translational status following AA treatment. To allow polysome profiling and poly(A) interactome capture of the same culture, prior to or following shift from 2% sucrose to equivalent media containing 0.02µg/ml AA (10 min), these were split. The result of two distinct biological experiments is shown. Association of FLAG-tagged eIF4A with mRNA was assessed by poly(A)-interactome capture and western blotting (top), and quantified by relative intensity normalized to poly(A) binding protein (Pab1) as a control for input and pull-down efficiency. Bar graph shows the amount of eIF4A bound to mRNA as a percentage of the pre-AA treatment level, the corresponding polysome gradient analyses are shown (grey box) with pre-treatment in scarlet and +10m AA in peach.

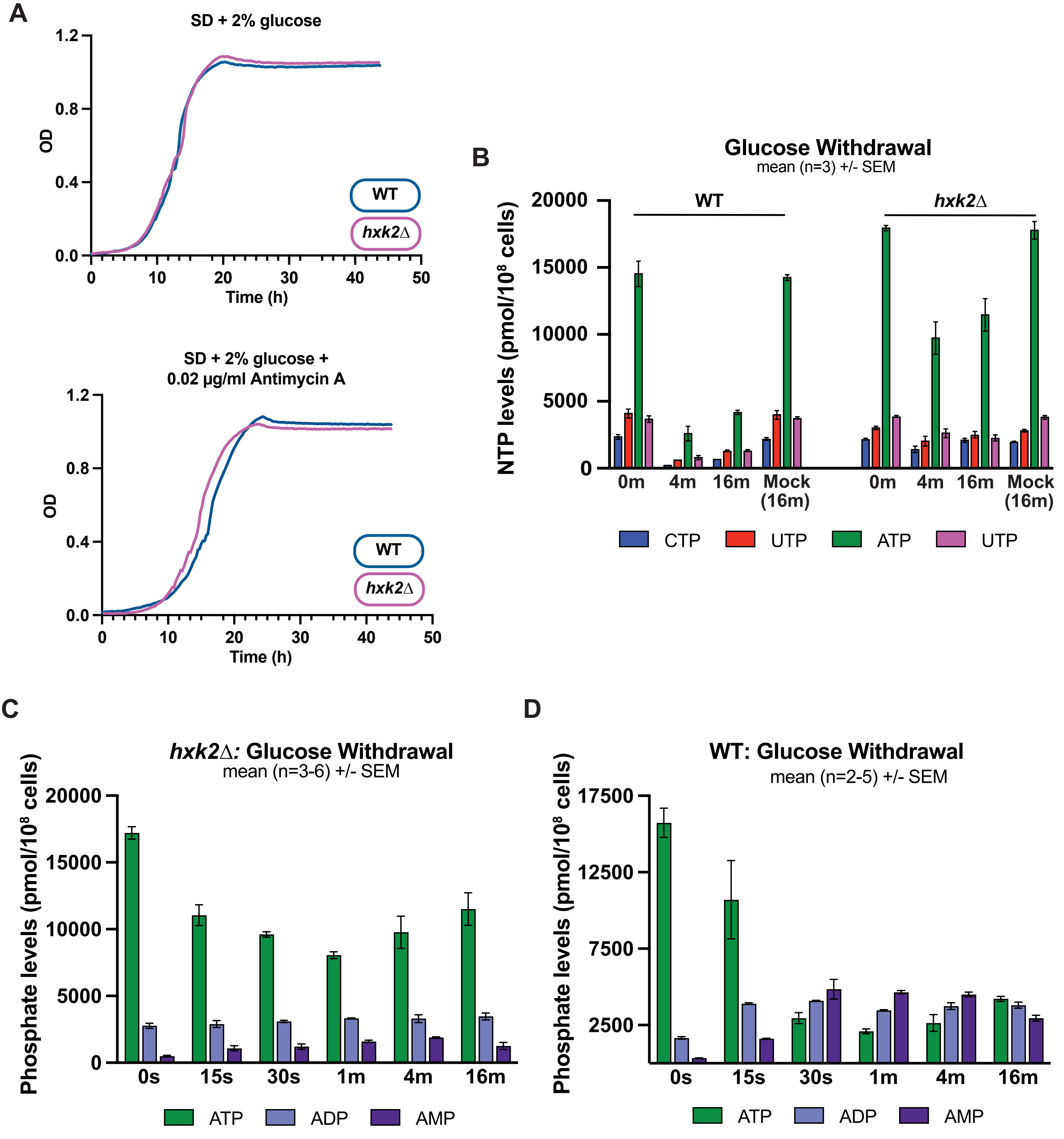

Supplementary Figure 2

Supplementary Figure 2. Nucleotide levels in wildtype and strains lacking Hxk2 (related to Figure 2)

A: Growth curves comparing growth of wild-type (WT, blue) and *hxk2*Δ (pink) BY4741 yeast strains. Growth rate on 2% glucose (SD) alone (upper) and following addition of 0.02μg/ml Antimycin A to exponential cultures (lower) was assessed by change in optical density (OD) over 48 hours. n=2.

B: Bar chart showing NTP levels measured following glucose withdrawal over minute time-courses in wild type (WT) and *hxk2*Δ strains. Replicate samples (n=3) were obtained at 0 min, 4 min, and 16 min following shift from 2% glucose to 2% glycerol/ethanol or a 16 min mock shift back to glucose. Error bars represent the mean +/− standard error (SEM).

C: Bar chart showing levels of intracellular adenosine phosphates in *hxk2*Δ yeast (monophosphate, AMP; diphosphate, ADP; triphosphate, ATP). Samples were obtained at 0 min, 15 sec, 30 sec, 1 min, 4 min and 16 min following shift from 2% glucose to 2% glycerol/ethanol or a 16 min mock shift back to glucose. Each condition has 6 replicates, except for 15 sec where n=3.

D: As in C for WT. Each condition has 5 replicates, expect for 15 sec where n=2. Error bars represent the mean +/− standard error (SEM).

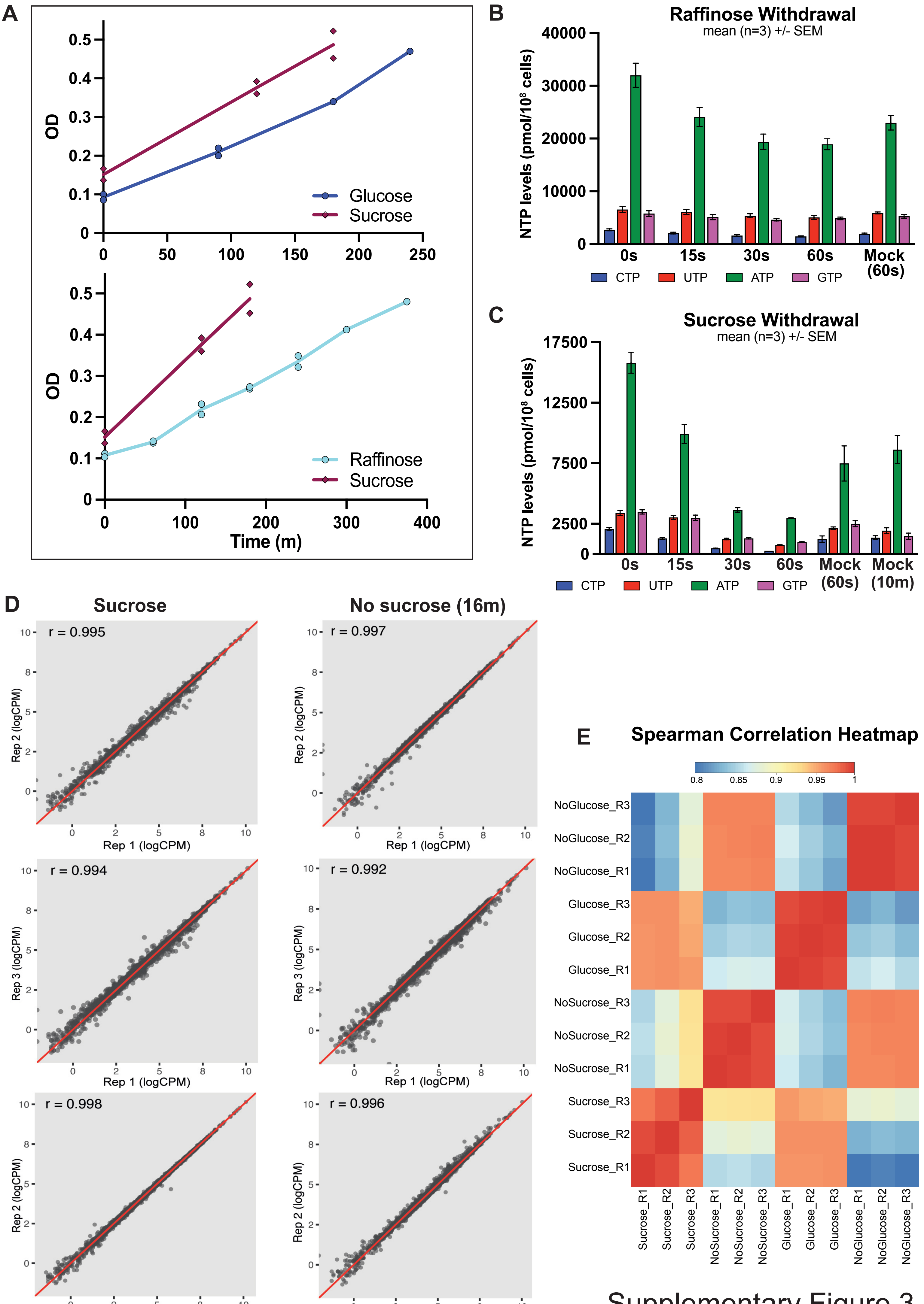

Supplementary Figure 3

Supplementary Figure 3. **Comparison of withdrawal of different carbon sources** (related to Figure 3)

A: Comparison of growth rate in yeast utilizing 2% sucrose relative to 2% glucose (upper) or 2% raffinose (lower). Growth was assessed by change in optical density (OD) during the exponential phase (from OD_{600} 0.1 - 0.5). n=2.

B: Bar chart showing NTP levels measured following raffinose withdrawal in *S.cerevisiae*. Replicate samples (n=3) were obtained at 15 sec, 30 sec and 1 min following shift from 2% raffinose to 2% glycerol/ethanol or a mock shift back to raffinose media. Error bars represent the mean +/− standard error (SEM). Mock shifts resulted in mild ATP depletion, but levels remain well above the Km for eIF4A after 60 sec

C: Bar chart showing NTP levels measured following sucrose withdrawal in *S.cerevisiae*. Replicate samples (n=3) were obtained at 15 sec, 30 sec and 1 min following shift from 2% sucrose to 2% glycerol/ethanol. Mock shift samples were harvested equivalently and returned to sucrose media for indicted times. Error bars represent the mean +/− standard error (SEM).

D: Scatter plots comparing replicate RNAseq datasets for sucrose grown and sucrose starved (16 min) *S.cerevisiae.* Counts are normalized for library depth as counts per million (CPM), all mRNAs for which > 15 sequence counts were recovered across 3 replicates are displayed. The correlation coefficient (r) for each replicate pair is shown.

E: Heatmap showing spearman correlation of sucrose withdrawal RNAseq datasets, with equivalent available data for glucose withdrawal [S1]. Samples were obtained prior-to or following shift from media containing either 2% glucose or sucrose to 2% glycerol/ethanol for 16 min (NoGlucose and NoSucrose, respectively). Spearman correlations between RNAseq data replicates were calculated using all mRNAs for which > 15 sequence reads were recovered (n=5,028).

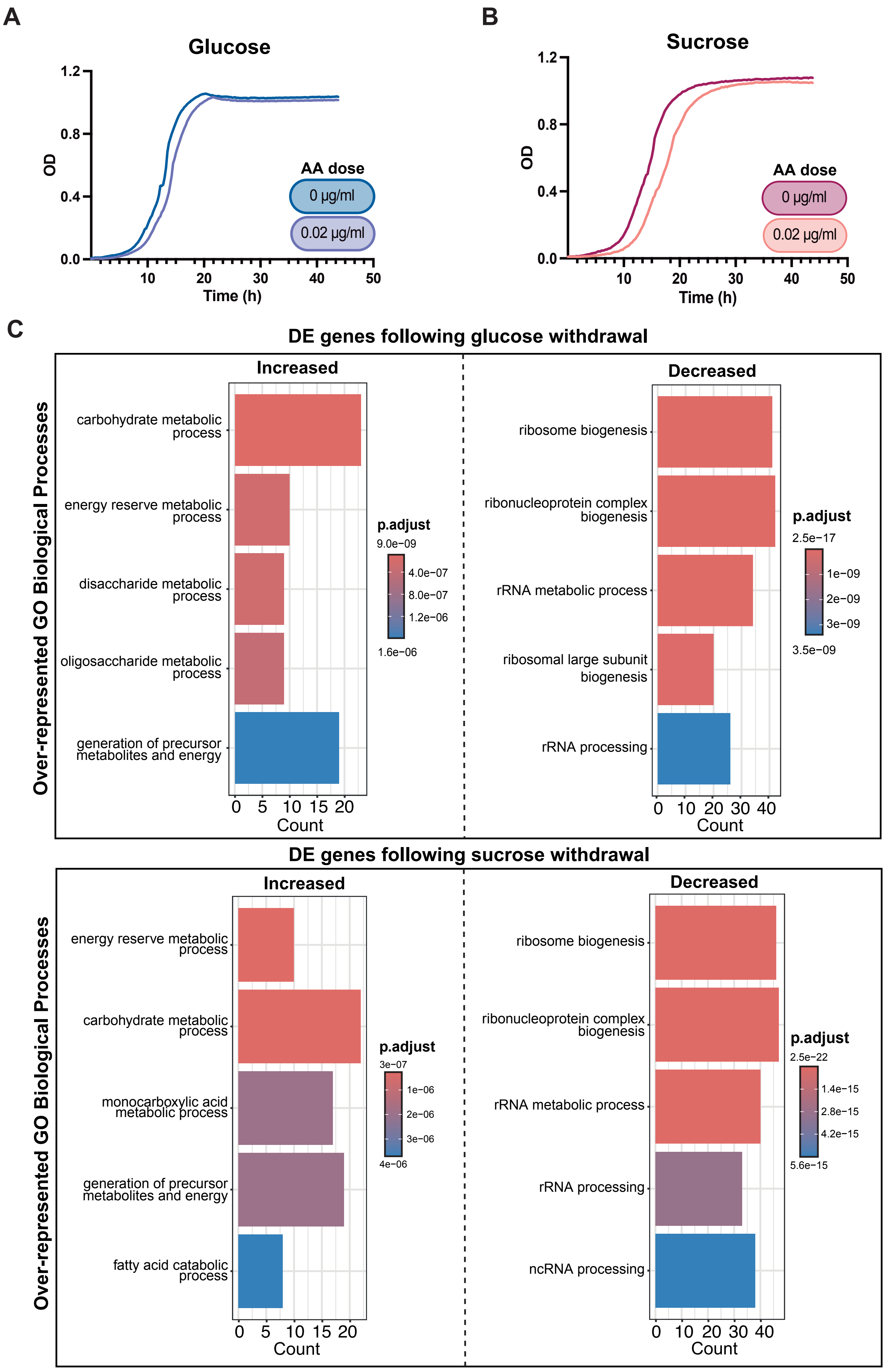

Supplementary Figure 4

Supplementary Figure 4. Transcription following glucose or sucrose withdrawal (related to Figure 4)

A: Growth curves comparing sensitivity to the mitochondrial inhibitor Antimycin A (AA) in yeast grown on glucose. The effect of the addition of 0.02μg/ml AA to the growth rate of exponential cultures was assessed by change in optical density (OD) over 48 hours as compared to mock treated cells (0μg/ml). n=2.

B: As in A, for yeast utilizing sucrose as carbon source.

C: Functional analysis of transcriptional changes following withdrawal of glucose (top) or sucrose (bottom) as carbon source. Gene ontology (GO) over-representation analysis was performed on the 100 transcripts with the greatest fold-change in expression (FDR <0.05) following withdrawal in either case. The 5 most enriched biological processes are shown, ordered and colored by significance of enrichment, given by adjusted p value (p.adjust). The number of transcripts annotated to each term is displayed (Count).

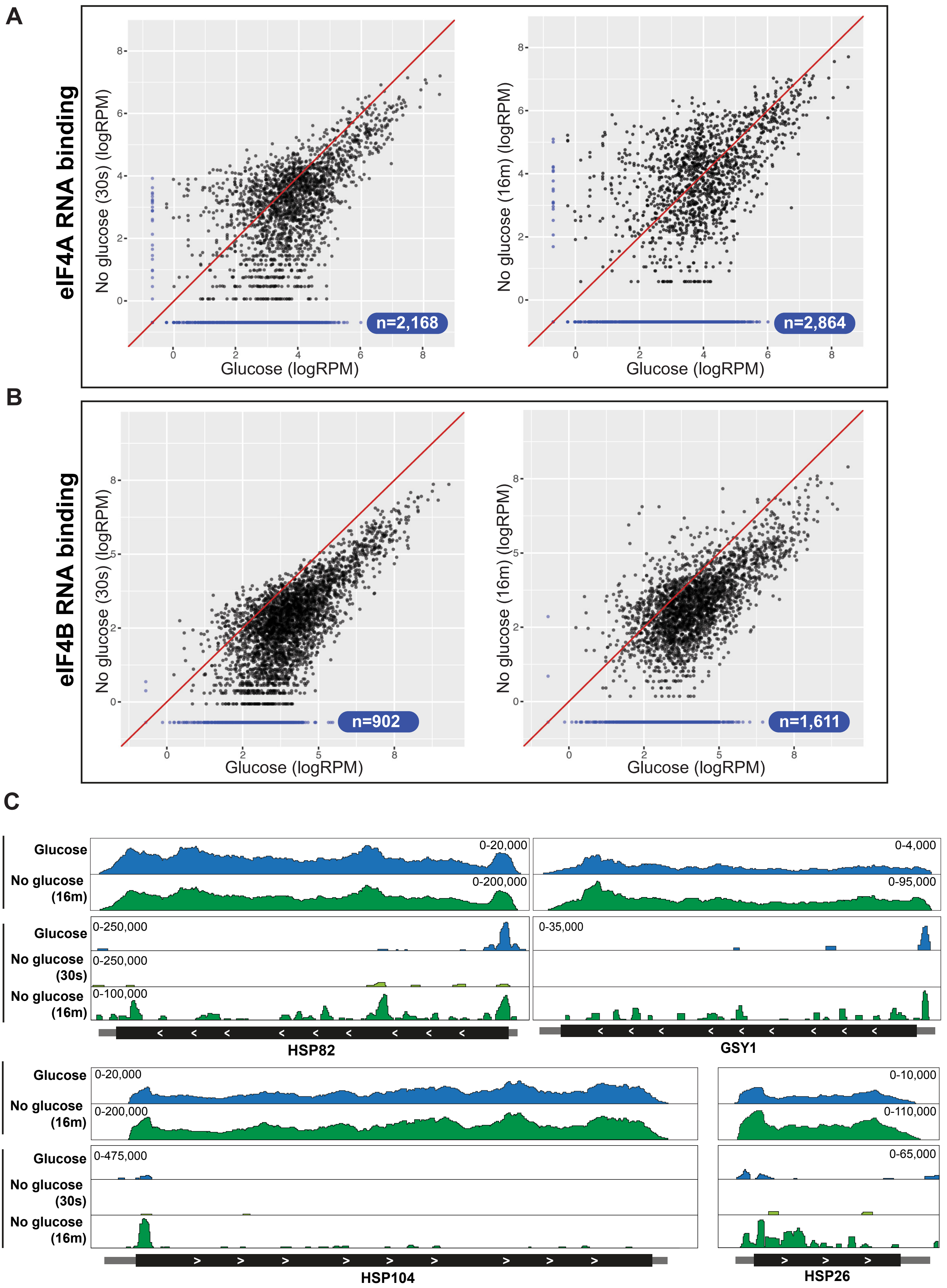

Supplementary Figure 5

Supplementary Figure 5. Binding of eIF4A and eIF4B after stress (related to Figure 5)
A: Scatter plots comparing mRNA binding by eIF4A following either 30 sec (left) or 16 min (right) glucose withdrawal to glucose replete conditions. Transcript counts are normalized to library size, in Reads per Million (RPM), and filtered for RPM > 0.5 in all the replicates of at least one condition (n =4,377). For transcripts detected in only one condition, imputation of the minimum threshold (RPM =0.5) was used, these are colored blue and ringed, with population size indicated.
B: As in A, for eIF4B
C: Examples of eIF4B binding across transcripts induced following glucose withdrawal (HSP82, GSY1, HSP104, HSP26), as in 5F.

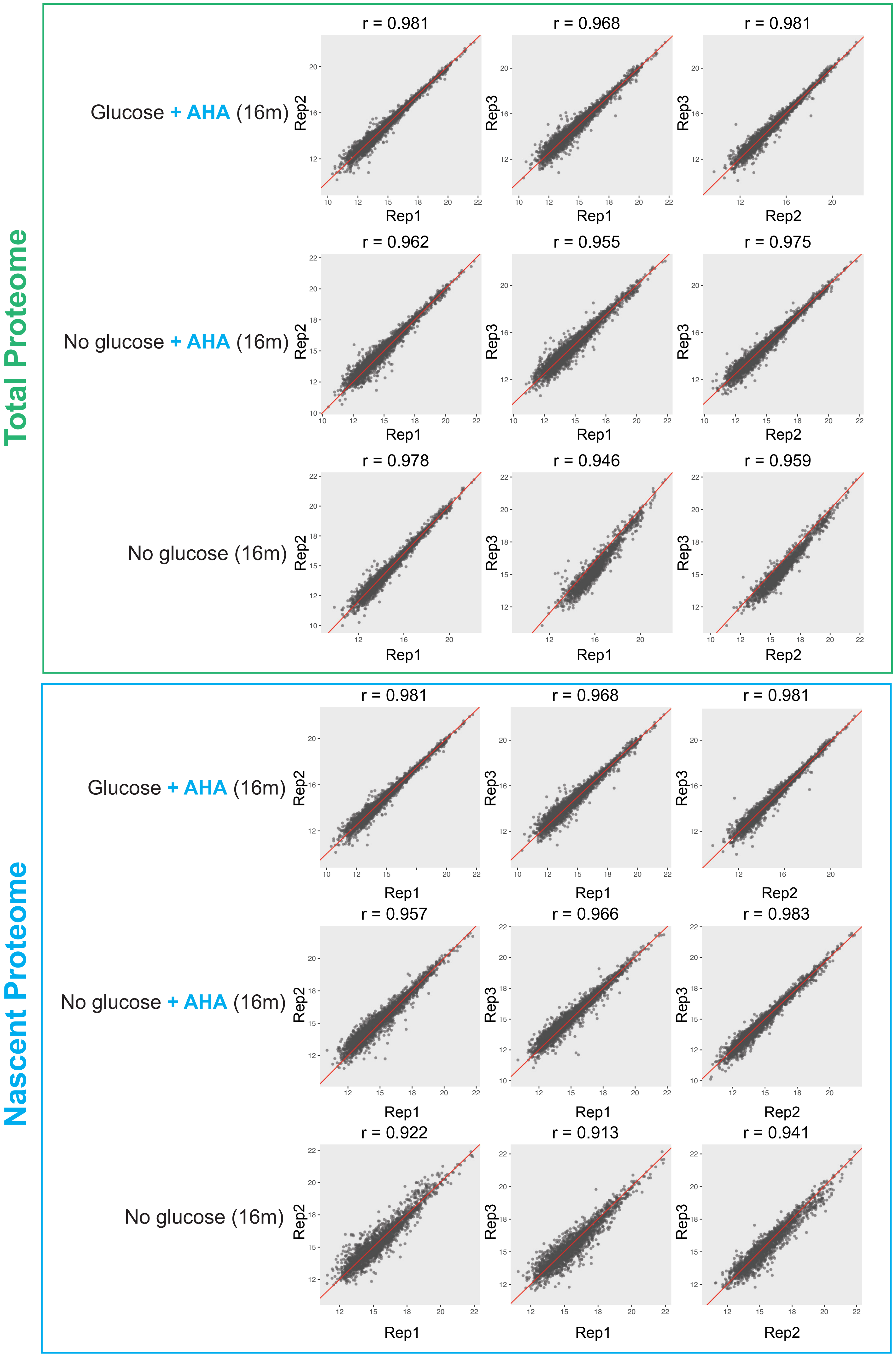

Supplementary Figure 6

Supplementary Figure 6. Comparison of proteome labeling replicates (related to Figure 6)

Scatter plots comparing replicate proteomic data sets. Yeast cultures were labelled using the methionine analogue L-Azidohomoalanine (AHA) for 16m, either directly in 2% glucose media or alongside shift to 2% glycerol/ethanol (No glucose). Unlabeled controls were prepared by transfer to media lacking AHA for the equivalent time. Nascent proteomes (blue box) were obtained by purification of AHA-labelled proteins see Figure 6a), while total proteomes reflect the inputs (green box). Protein intensities are log2 normalized, and the correlation coefficient (r) for each replicate pair is shown.

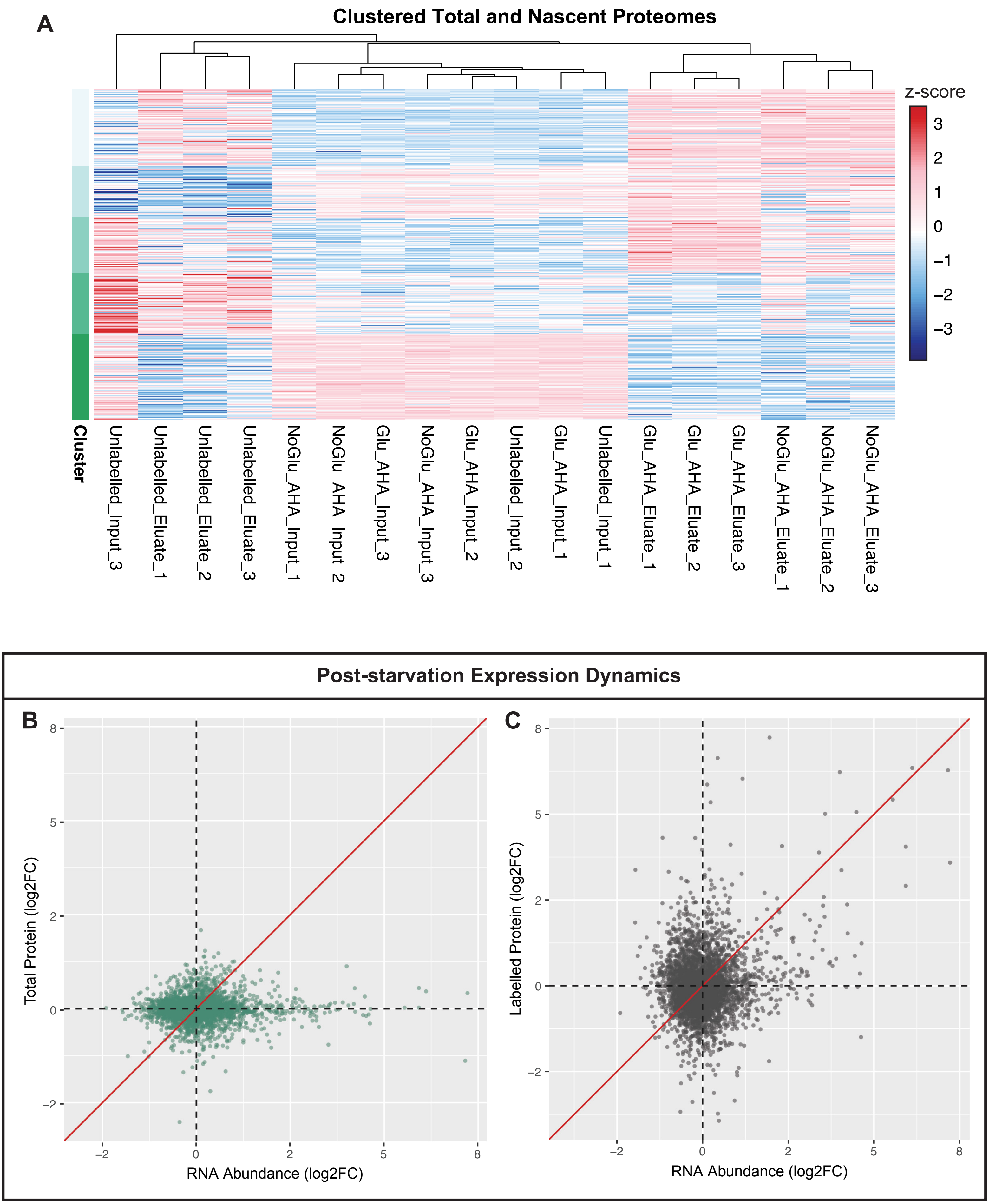

Supplementary Figure 7

Supplementary Figure 7. Clustering of nascent proteomes (related to Figure 7)
A: Heatmap for all proteomes analyzed (as described S6), comparing quantified protein intensity by z-score (deviation from average). K-means clustering was used to define sets of proteins with similar expression profiles (k = 5, 100 rounds) (rows). Within these groups, samples were ordered by hierarchical clustering, visualized by accompanying dendrogram (columns).
B: Scatter plots comparing fold change (FC) in abundance (RPKM) of a transcript following 16 min glucose withdrawal, to the level of the associated protein in the nascent (labelled, left) and total (right) proteome. Fold change (FC) in log2 normalized protein intensities are plotted, and the x=y line is shown in red.

SUPPLEMENTARY TABLES

Data Set	Purpose	Repository	Samples Used
CRAC sequence data (Bresson et al., 2020)	eIF4A binding<br>Analysis of mechanism for selective translation following glucose withdrawal	GSE148166	GSM4455414<br>GSM4455411<br>GSM4455368<br>GSM4455375<br>GSM4455413<br>GSM4455416<br>GSM4455369<br>GSM4455376<br>GSM4455385
CRAC sequence data (Bresson et al., 2020)	eIF4B binding<br>Analysis of mechanism for selective translation following glucose withdrawal	GSE148166	GSM4455366<br>GSM4455378<br>GSM4455382<br>GSM4455406<br>GSM4455407<br>GSM4455408<br>GSM4455379<br>GSM4455383<br>GSM4455367
RNA sequencing data (Ristova et al., 2024)	BY4741 Transcript expression<br>Analysis in changes in expression of mRNA following glucose withdrawal	GSE283345	GSM8660580<br>GSM8660581<br>GSM8660582<br>GSM8660586<br>GSM8660587<br>GSM8660588
RNA sequencing data (This study)	BY4741 Transcript expression<br>Analysis in changes in expression of mRNA following sucrose withdrawal	GSE285035	GSM8695551<br>GSM8695552<br>GSM8695553<br>GSM8695554<br>GSM8695555<br>GSM8695556

Table S1: (Related to STAR Methods) Datasets and links used in this work.

WT: Glucose -> Glyc/EthOH						
Time (m)	Concentration					
	ATP	GTP	CTP	UTP	ADP	AMP
0	1 957229	0 455529	0 301959	0 51596	0 1843157	0 0378775
0 25	1 125253	0 331191	0 190317	0 412057	0 4342126	0 1797485
0 5	0 297651	0 118048	0 058094	0 152139	0 4546362	0 5391429
1	0 228918	0 087803	0 026698	0 08459	0 3844503	0 5167863
4	0 293253	0 092691	0 028546	0 072796	0 4153788	0 5006227
16	0 468443	0 146508	0 077965	0 146253	0 4228738	0 328312

WT: Sucrose -> Sucrose + 0.02µg/ml Antimycin A				
Time (m)	Concentration (mM)			
	ATP	GTP	CTP	UTP
0	1 8034956	0 3898235	0 2502403	0 426058
0 25	1 359935	0 3388231	0 17609	0 3554581
0 5	1 2151579	0 3093561	0 1424872	0 3136151
1	1 1480898	0 2857888	0 1304686	0 294109
4	0 0952548	0 0559064	0 0151773	0 0387523
16	0 2913878	0 085451	0 0779138	0 1483645

hxk2Δ : Glucose -> Glyc/EthOH						
Time (m)	Concentration (mM)					
	ATP	GTP	CTP	UTP	ADP	AMP
0	1 9120949	0 3499594	0 2155984	0 3218635	0 3077448	0 0555322
0 25	1 2271558	0 304753	0 1391853	0 2848869	0 3216902	0 1203092
0 5	1 067046	0 2767799	0 1192916	0 2522272	0 3434638	0 133677
1	0 8951032	0 2459794	0 0951211	0 1859587	0 3703437	0 1770802
4	1 085439	0 297991	0 1609335	0 230493	0 3669536	0 2095575
16	1 278784	0 252026	0 237948	0 278415	0 3854136	0 139737

WT: Sucrose -> Glyc/EthOH				
Time (m)	Concentration (mM)			
	ATP	GTP	CTP	UTP
0	1 7563475	0 3865426	0 2313018	0 3776374
0 25	1 1016295	0 3320449	0 1434634	0 3363138
0 5	0 4058269	0 1438098	0 0529269	0 137581
1	0 3296063	0 1101411	0 0299628	0 083342

WT: Raffinose -> Glyc/EthOH

Time (m)	Concentration (mM)			
	ATP	GTP	CTP	UTP
0	3 5550875	0 6425616	0 3011699	0 7280659
0 25	2 6765842	0 5673495	0 2312573	0 6747306
0 5	2 1537432	0 5141217	0 1810623	0 5952718
1	2 1021956	0 5420719	0 1618283	0 5601914

pin4Δ : Glucose -> Glyc/EthOH				
Time (m)	Concentration (mM)			
	ATP	GTP	CTP	UTP
0	1 3208329	0 3071416	0 2206757	0 4178835
0 25	0 7238615	0 2094109	0 1290003	0 2848244
0 5	0 1637364	0 0642338	0 0379532	0 1001792
1	0 1281127	0 0452382	0 0203295	0 0577417

Supplementary Table 2: (Related to STAR Methods and Figures 1, 2, and 3) Quantified NTP Levels Reported as Estimated Concentrations Average of 3 replicates in pmol/cell, then converted to intracellular concentrations (mM) as described in methods.

Supplementary Reference;

[S1] Ristová, M., Bexley, K., Shchepachev, V., Cook, A.G., and Tollervey, D. (2024). Pin4 Links Post-transcriptional and Transcriptional Responses to Glucose Starvation in Yeast. Preprint at bioRxiv, https://doi.org/10.1101/2024.11.13.623376
